# Primary rhabdomyosarcoma of the ethmoid sinus with orbital extension and metastasis to the pancreatic body

**DOI:** 10.1002/ccr3.4149

**Published:** 2021-05-04

**Authors:** Hiroaki Itamochi, Hisanori Ariga, Kiyoto Shiga, Noriyuki Uesugi, Tamotsu Sugai

**Affiliations:** ^1^ Department of Clinical Oncology Iwate Medical University School of Medicine Yahaba‐Cho Japan; ^2^ Department of Radiation Oncology Iwate Medical University School of Medicine Yahaba‐Cho Japan; ^3^ Department of Head and Neck Surgery Iwate Medical University School of Medicine Yahaba‐Cho Japan; ^4^ Department of Molecular Diagnostic Pathology Iwate Medical University School of Medicine Yahaba‐Cho Japan

**Keywords:** adult, ethmoid sinus, metastases, pancreas, rhabdomyosarcoma

## Abstract

This case report highlights the need for clinicians to monitor patients with rhabdomyosarcoma for pancreatic metastasis to ensure that proper treatment is quickly provided, thereby improving outcomes.

## Graphical abstract

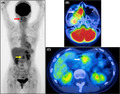

## 1 INTRODUCTION

Rhabdomyosarcoma (RMS) is a rare cancer in adults, and metastasis to the pancreas is uncommon. We have reported a case of adult ethmoid sinus RMS with orbital extension and metastasis to the pancreatic body. The patient received radiotherapy and chemotherapy, and the tumors were completely ablated by this regimen.

RMS is a malignant tumor of mesenchymal origin and is believed to arise from primitive skeletal muscle cells of various anatomical sites.^1^ It is the most common soft tissue sarcoma in childhood and adolescence but rarely presents in adulthood.^2^ Although RMS may occur at any age, 41% of all cases occur in adults, comprising 1% of adult cancers.^2^, ^3^ The primary tumor site of adult RMS has a wide anatomic distribution, with most tumors classified as visceral.^3^, ^4^ Tumors of the head and neck only account for 19%‐24% of adult RMS cases.^3^, ^4^ Adult RMS is an aggressive tumor, and more than half of patients have regional and distant metastases at diagnosis.^3^, ^4^ The common sites of metastasis include the lungs, bone, omentum, and lymph nodes.^5^ Pancreatic metastases are rare in newly diagnosed patients.^6^, ^7^

The prognosis of adult RMS is dismal, especially in patients with distant metastasis.^3^, ^4^, ^5^, ^8^ Although standard therapies have not been established, surgery remains the mainstay of treatment, and complete resection should be attempted whenever possible. For patients with residual or recurrent disease, chemotherapy, and/or radiotherapy may be reasonable options.^4^, ^5^ In this study, we have reported an adult case of RMS of the ethmoid sinus with orbital extension and metastasis to the pancreatic body in which a complete response was achieved following combination treatment with radiotherapy and chemotherapy.

## 2 CASE REPORT

**FIGURE 1:**
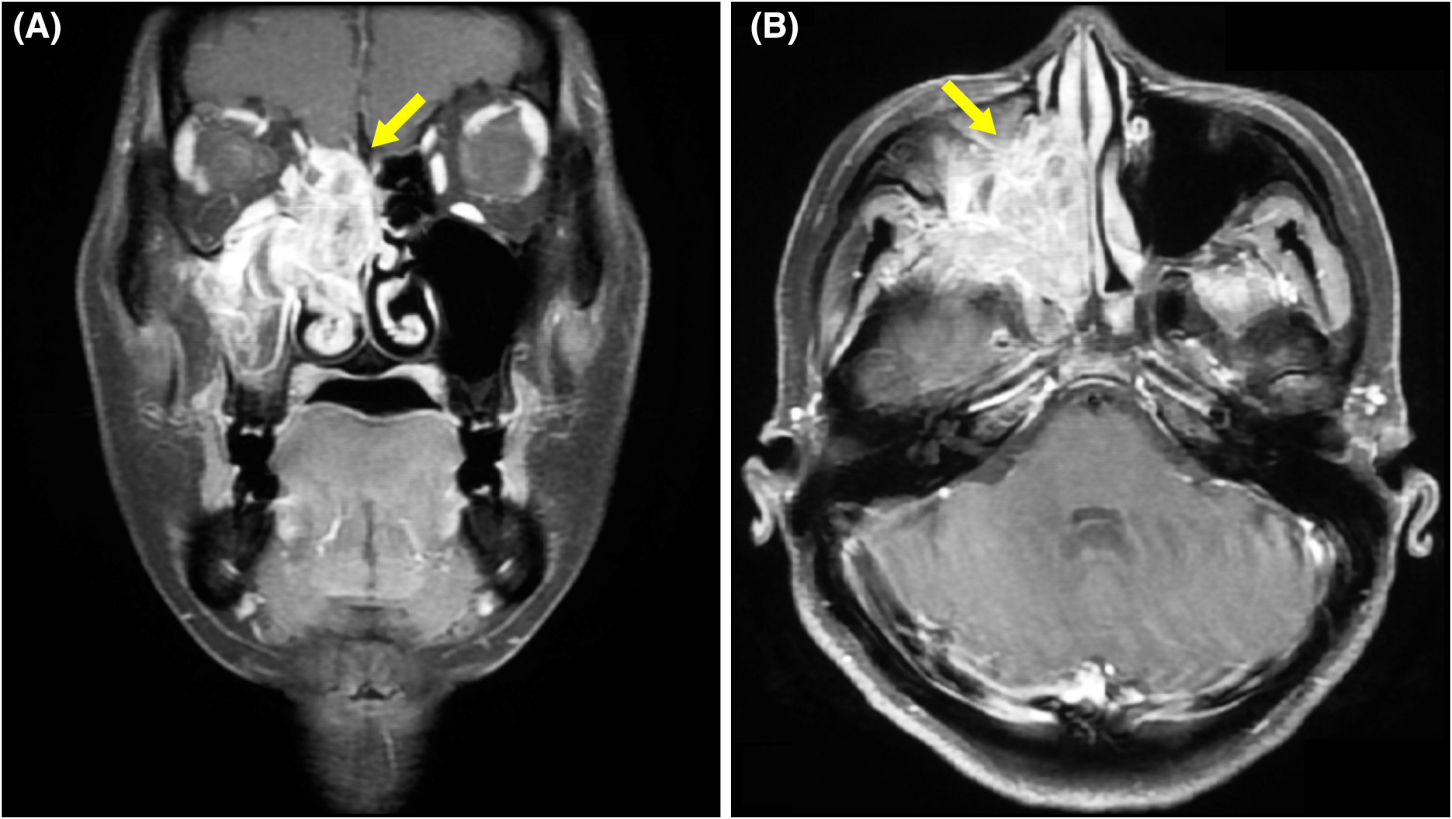
Coronal (A) and axial (B) magnetic resonance imaging of the head revealing a large homogenous well‐enhanced mass (42 × 21 mm) in the right ethmoid sinus (yellow arrow). The mass had extended to the right sphenoid sinus, nasal cavity, and orbit

**FIGURE 2:**
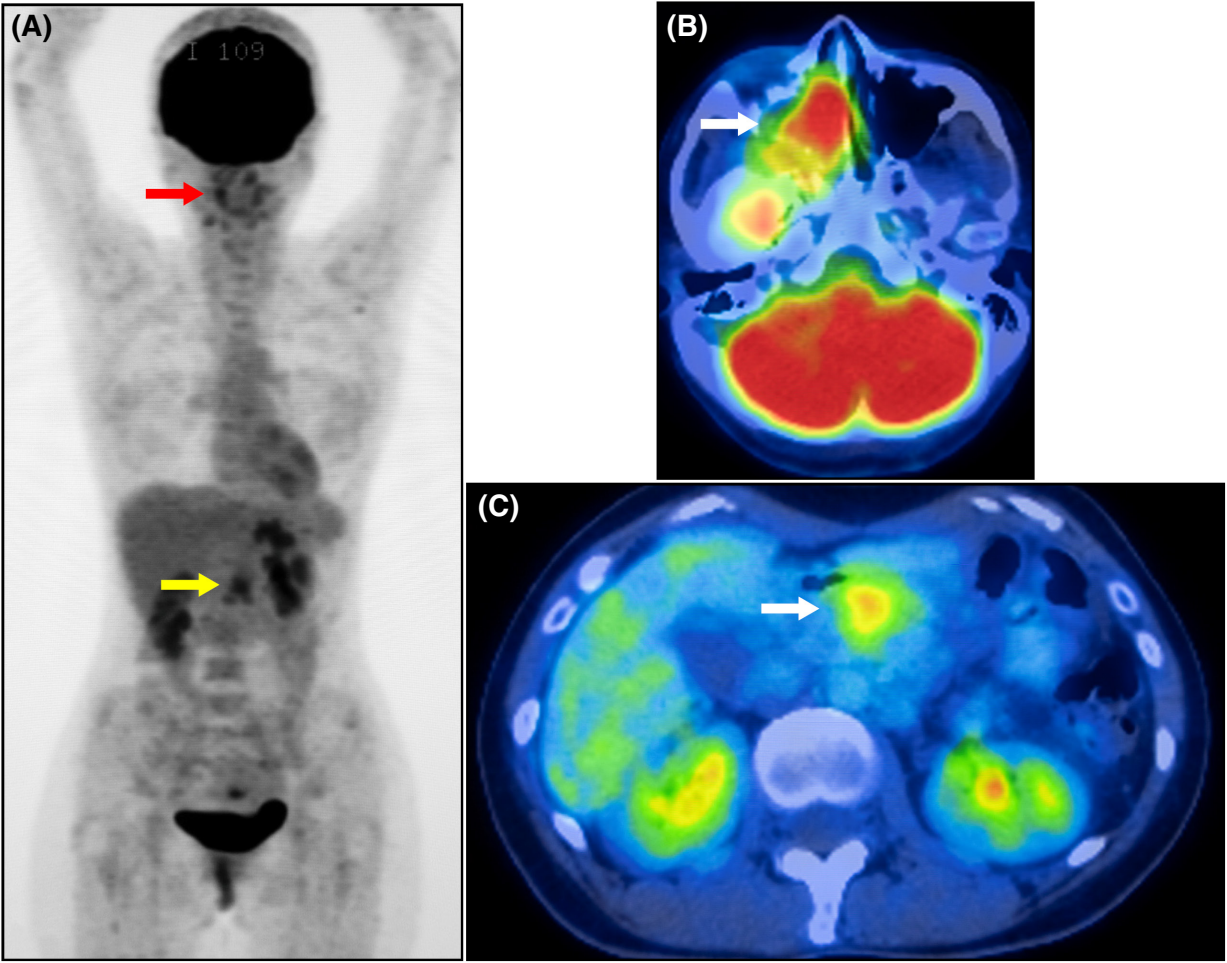
Positron emission tomography‐computed tomography. (A) The maximum intensity projection image of the whole body revealed increased tracer uptake in the right perinasal region (red arrow) and midabdomen (yellow arrow). Transaxial‐fused images revealed hypermetabolic masses (white arrows) in the right ethmoid sinus (B) and pancreatic body (C)

**FIGURE 3:**
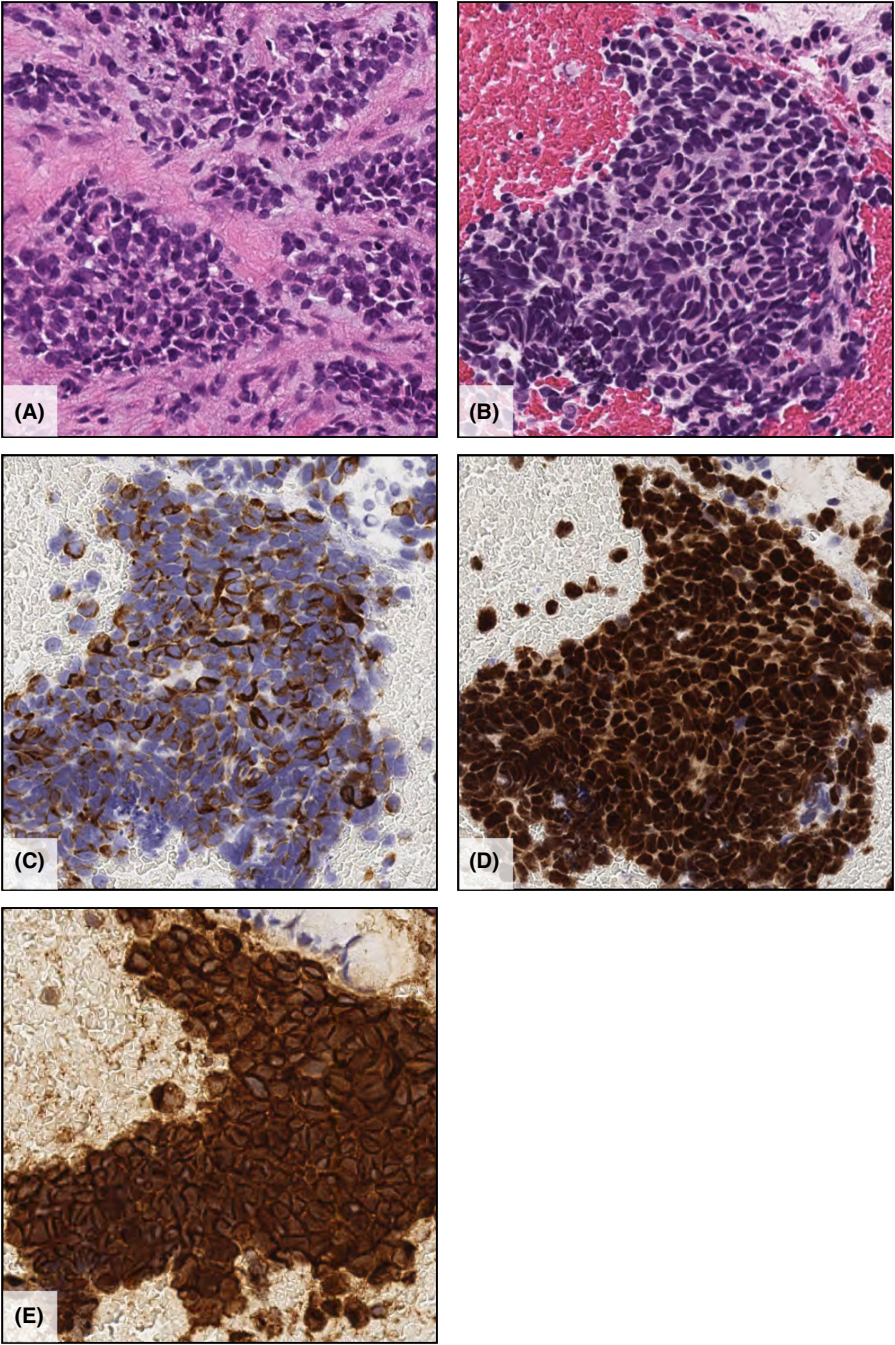
Microscopic examination of the right ethmoid sinus tumor (A) and pancreatic tumor (B). The small round malignant cells were arranged in nests and sheets and surrounded by fibrous stroma (hematoxylin and eosin staining, ×400). On immunohistochemical evaluation, the tumor cells were positive for desmin (C), MyoD1 (D), and vimentin (E)

The patient was a 44‐year‐old Japanese woman (gravida 1 para 1) who presented at a local hospital with upper right gum pain and nose bleeding. Computed tomography (CT) revealed a 35 × 20 mm irregular mass in the right ethmoid sinus. Biopsy of the nasal mass was performed, revealing small round to oval malignant cells arranged in varying degrees of cellularity. She was subsequently referred to our clinic. Magnetic resonance imaging disclosed a large homogenous well‐enhanced mass (42 × 21 mm) with surrounding bony erosion and remodeling (Figure 1). The mass had extended to the right sphenoid sinus, nasal cavity, and orbit. Although the corrected visual acuity was 1.0 in the left eye, the right eye displayed complete vision loss. Ptosis and oculomotor nerve palsy were also observed in the right eye. Positron emission tomography (PET)‐CT revealed an enhanced mass in the right ethmoid sinus and pancreatic body (Figure 2). Biopsy of the pancreatic tumor revealed a small round malignant cell tumor. Immunohistochemically, both nasal and pancreatic tumors were positive for desmin, MyoD1, and vimentin (Figure 3) but were negative for cytokeratin AE1/AE3, CK7, CK20, synaptophysin, chromogranin A, NSE, LCA, CD3, CD4, CD8, CD10, CD20, CD79a, S100, trypsin, MIC2, CAM5.2, calretinin, and Bcl‐10. The Ki‐67 labeling index was 80%. Therefore, the pathological diagnosis was embryonal RMS of the ethmoid sinus with orbital extension and metastasis to the pancreatic body.

The treatment strategy was radiotherapy (50 Gy) of the ethmoid sinus and systemic chemotherapy consisting of vincristine 1.5 mg/m^2^ on days 1, 8, and 15, actinomycin D 0.045 mg/kg, and cyclophosphamide 1200 mg/m^2^ (VAC) administered at 3‐week intervals. After irradiation of the ethmoid sinus, 45 Gy of radiotherapy was delivered to the pancreatic body. After first cycle of VAC therapy, the patient developed febrile neutropenia. Then, the doses were decreased to vincristine 1.2 mg/m^2^, actinomycin D 0.036 mg/kg, and cyclophosphamide 1000 mg/m^2^. However, after a second cycle of VAC therapy, she developed grade 4 leukocytopenia. Thereafter, the doses were further reduced to vincristine 0.9 mg/m^2^, actinomycin D 0.027 mg/kg, and cyclophosphamide 720 mg/m^2^. The patient completed a total of 14 cycles of VAC therapy. CT revealed complete regression of the previous tumors. She has remained alive and healthy for 15 months since starting radiotherapy and VAC therapy.

## 3 DISCUSSION

Malignant tumors of the paranasal sinuses comprise <1% of all malignancies, and sarcomas of the paranasal sinuses represent approximately 7% of all head and neck sarcomas.^9^, ^10^ Forty‐six percent of paranasal sinus sarcomas were the RMS type in adults, and the maxillary sinus was the most frequent primary site (50%) of RMS among paranasal sinuses, followed by the ethmoid sinus (35%).^11^ Considering patients with sarcoma of the ethmoid sinus, the most common tumor histology was RMS (68%).^11^

Although the 5‐year overall survival (OS) rate of localized RMS exceeds 70% in children (<18), the prognosis of adult RMS is extremely poor.^3^, ^4^, ^5^, ^8^ Other important prognostic factors include tumor size, the presence of metastases, and histological subtypes.^8^ Bompas et al reported 5‐year OS rates for localized RMS and advanced RMS (with nodes and/or metastases) of 43% and 5% in adults, respectively.^8^ Patients with metastatic disease have poorer prognoses, and they should be considered for various treatment approaches, highlighting the need for accurate staging. Full initial staging employs cross‐sectional imaging of the primary tumor, chest, abdomen, and pelvis; bone scan; and pelvic bone marrow biopsies. Several studies have shown that PET‐CT improved the initial staging accuracy in adult RMS, specifically the detection of distant metastatic spread.^6^, ^12^, ^13^ In our patient, pancreatic body metastasis was detected by PET‐CT.

The pancreas is considered a rare metastatic site in patients with newly diagnosed RMS.^6^, ^7^ In a retrospective analysis, four (5.6%) of 77 patients with alveolar RMS had pancreatic metastases at initial presentation, including one adult case, whereas four patients presented with pancreatic metastases at the time of disease recurrence, including one adult case.^7^ In the two adult patients with RMS and pancreatic metastases, the primary tumor locations were the face (right orbit and periorbital region) in one patient and the left upper extremity (left shoulder region) in the other. Even though pancreatic metastasis is rare in adult RMS, evaluation of the pancreas might be needed in this disease.

The management of patients with RMS involves the surgery and/or radiotherapy for local control and chemotherapy of various intensity and duration depending on the risk group of assignment. Systemic therapy consisting of an alkylating agent (ie, cyclophosphamide or ifosfamide) combined with vincristine and actinomycin D administered every 3 weeks for 6‐10 months is the standard backbone therapy for patients with intermediate‐ or high‐risk RMS.^14^ Therefore, we used VAC therapy as the first‐line chemotherapy in the present case. During VAC therapy, our patient underwent radiotherapy of the primary lesion (ethmoid sinus) and pancreatic metastasis. Although our patient has survived for 15 months after the start of radiotherapy and VAC therapy, close follow‐up is needed to detect recurrence.

## 4 CONCLUSION

Adult RMS is extremely rare, and its prognosis is dismal, especially in patients with distant metastasis. PET‐CT may be useful for accurately diagnosing distant metastases of RMS. Pancreatic metastases are infrequent in adult patients with RMS but might need to be evaluated.

## CONFLICT OF INTEREST

The authors declare that they have no conflict of interest.

## AUTHOR CONTRIBUTIONS

HI: contributed to concepts and manuscript editing. HA and KS: contributed to design. HA, KS, and NU: contributed to definition of intellectual content. HI, HA, KS, and NU: contributed to manuscript editing. TS: contributed to literature search and manuscript review.

## ETHICAL APPROVAL

This was conducted ethically in accordance with the World Medical Association Declaration of Helsinki. Patients have given their written informed consent.

## PATIENT'S CONSENT

Yes.

## Data Availability

The data that support the findings of this study are available from the corresponding author upon reasonable request.

## References

[1] Dagher R , Helman L . Rhabdomyosarcoma: an overview. Oncologist. 1999;4:34‐44. 10337369

[2] Amer KM , Thomson JE , Congiusta D , et al. Epidemiology, incidence, and survival of rhabdomyosarcoma subtypes: SEER and ICES database analysis. J Orthop Res. 2019;37:2226‐2230. 31161653 10.1002/jor.24387

[3] Sultan I , Qaddoumi I , Yaser S , Rodriguez‐Galindo C , Ferrari A . Comparing adult and pediatric rhabdomyosarcoma in the surveillance, epidemiology and end results program, 1973 to 2005: an analysis of 2,600 patients. J Clin Oncol. 2009;27:3391‐3397. 19398574 10.1200/JCO.2008.19.7483

[4] Hawkins WG , Hoos A , Antonescu CR , et al. Clinicopathologic analysis of patients with adult rhabdomyosarcoma. Cancer. 2001;91:794‐803. 11241248

[5] Ferrari A , Dileo P , Casanova M , et al. Rhabdomyosarcoma in adults. A retrospective analysis of 171 patients treated at a single institution. Cancer. 2003;98:571‐580. 12879475 10.1002/cncr.11550

[6] Natarajan A , Puranik A , Purandare N , Agrawal A , Shah S , Rangarajan V . An infrequent case of adult alveolar rhabdomyosarcoma with pancreatic metastases detected in F‐18 FDG PET/CT. Indian J Nucl Med. 2017;32:227‐229. 28680211 10.4103/ijnm.IJNM_28_17 PMC5482023

[7] Jha P , Frolich AM , McCarville B , et al. Unusual association of alveolar rhabdomyosarcoma with pancreatic metastasis: emerging role of PET‐CT in tumor staging. Pediatr Radiol. 2010;40:1380‐1386. 20180103 10.1007/s00247-010-1572-3 PMC2895865

[8] Bompas E , Campion L , Italiano A , et al. Outcome of 449 adult patients with rhabdomyosarcoma: an observational ambispective nationwide study. Cancer Med. 2018;7:4023‐4035. 29956493 10.1002/cam4.1374 PMC6089183

[9] Weber AL , Stanton AC . Malignant tumors of the paranasal sinuses: radiologic, clinical, and histopathologic evaluation of 200 cases. Head Neck Surg. 1984;6:761‐776. 6319335 10.1002/hed.2890060310

[10] Wanebo HJ , Koness RJ , MacFarlane JK , et al. Head and neck sarcoma: report of the head and neck sarcoma registry. Society of head and neck surgeons committee on research. Head Neck. 1992;14:1‐7. 1624288 10.1002/hed.2880140102

[11] Martin E , Radomski S , Harley E . Sarcomas of the paranasal sinuses: an analysis of the SEER database. Laryngoscope Investig Otolaryngol. 2019;4:70‐75. 10.1002/lio2.245 PMC6383302 30828621

[12] Tateishi U , Hosono A , Makimoto A , et al. Comparative study of FDG PET/CT and conventional imaging in the staging of rhabdomyosarcoma. Ann Nucl Med. 2009;23:155‐161. 19225939 10.1007/s12149-008-0219-z

[13] Saboo SS , Krajewski KM , Zukotynski K , et al. Imaging features of primary and secondary adult rhabdomyosarcoma. AJR Am J Roentgenol. 2012;199:W694‐W703. 23169742 10.2214/AJR.11.8213

[14] Skapek SX , Ferrari A , Gupta AA , et al. Rhabdomyosarcoma. Nat Rev Dis Primers. 2019;5:1. 30617281 10.1038/s41572-018-0051-2 PMC7456566

